# Coagulation parameters are associated with the prognosis of immunoglobulin a nephropathy: a retrospective study

**DOI:** 10.1186/s12882-020-02111-1

**Published:** 2020-10-27

**Authors:** Ming Xia, Di Liu, Liang Peng, Yan Li, Haiyang Liu, Lingzhi Wu, Guochun Chen, Yu Liu, Hong Liu

**Affiliations:** 1 Department of Nephrology, The Second Xiangya Hospital, Central South University, 139 Renmin Road, Changsha, 410011 Hunan China; 2 Hunan Key Laboratory of Kidney Disease and Blood Purification, 139 Renmin Road, Changsha, 410011 Hunan China

**Keywords:** Coagulation, Fibrosis, IgA nephropathy, Prognosis

## Abstract

**Background:**

Interstitial fibrosis/tubular atrophy (T) score is a known determinant of the progression of immunoglobulin A nephropathy (IgAN). Strong evidence indicates that the components of the coagulation system closely linked with fibrotic events have been highlighted in the kidney. However, whether the coagulation system can affect the renal outcome of IgAN remains unclear. Herein, we investigated the association of coagulation parameters and pathological phenotype of IgAN and their combined effects on the deterioration of renal function.

**Methods:**

This retrospective study included *N* = 291 patients with biopsy-proven IgAN from May 2009 to April 2013 in the Second Xiangya Hospital. Clinical data, pathological features were collected, and the associations of coagulation parameters at biopsy, T score, and renal outcome were evaluated. T score indicated the degree of tubular atrophy or interstitial fibrosis. The renal outcome was defined as an end-stage renal disease (ESRD) or an irreversible 50% estimated glomerular filtration rate (eGFR) reduction.

**Results:**

Shorter prothrombin time (PT) and the activated partial thromboplastin time (APTT) were significantly associated with T (both *p* < 0.001). PT (< 11.15 s) or APTT (< 29.65 s) had worse cumulative survival rate (*p* = 0.008, *p* = 0.027 respectively) and were significantly but not independently associated with a higher risk of renal outcome (*p* = 0.012, *p* = 0.032 respectively). In the combined analyses of PT, APTT, and T lesions, the odd ratios for the outcome were significantly higher in the presence of T with PT (< 11.15 s) or APTT (< 29.65 s).

**Conclusion:**

Shorter PT and APTT are associated with an increased incidence of the T lesion and are additional factors that portend a poorer prognosis in IgAN. Monitoring coagulation function might be important when assessing the risk of progression. Additional studies exploring the molecular mechanism between coagulation and IgAN pathology are needed.

## Background

Immunoglobulin A nephropathy (IgAN) is one of the most prevalent primary glomerulonephritis worldwide, especially, accounting for ~ 40% in Asia [1], and ~ 50% in China [2]. It is the main cause of end-stage renal failure (ESRD), approximately 30–40% of IgAN patients develop ESRD, which brings a heavy burden to individuals and society [3]. Therefore, it is of great scientific and practical significance to identify the risk factors associated with the progression of IgAN. The oxford classification MEST-C score is well regarded prognostic indicators for IgAN, containing the presence of mesangial hypercellularity (M), endocapillary hypercellularity (E), segmental glomerulosclerosis (S), tubular atrophy/interstitial fibrosis (T), crescent (C) based on renal histopathology [4, 5]. Of them, the T score is the most valuable histological parameter, confirmed by a large number of original studies [6–8]. T lesion is not merely a histomorphological feature of IgAN but is rather a final common pathway for most progressive kidney diseases and leads to advanced chronic kidney disease (CKD) [9, 10]. However, factors associated with tubulointerstitial damage of IgAN have not been elucidated.

Given the potential role of the coagulation system in the development of renal fibrosis, the regeneration and proliferation of tubular or mesangial cells [11–13], it is interesting and clinically useful to explore whether coagulation parameters are linked to the pathological Oxford classification of IgAN. In this study, we sought to investigate two key concepts. First, are coagulation parameters at the time of renal biopsy associated with T score? Second, how much do the coagulation parameters affect the renal outcome? To investigate this, we identified a cohort of patients with biopsy-proven IgAN and collected their clinical and laboratory parameters.

## Methods

### Subjects

**Fig. 1:**
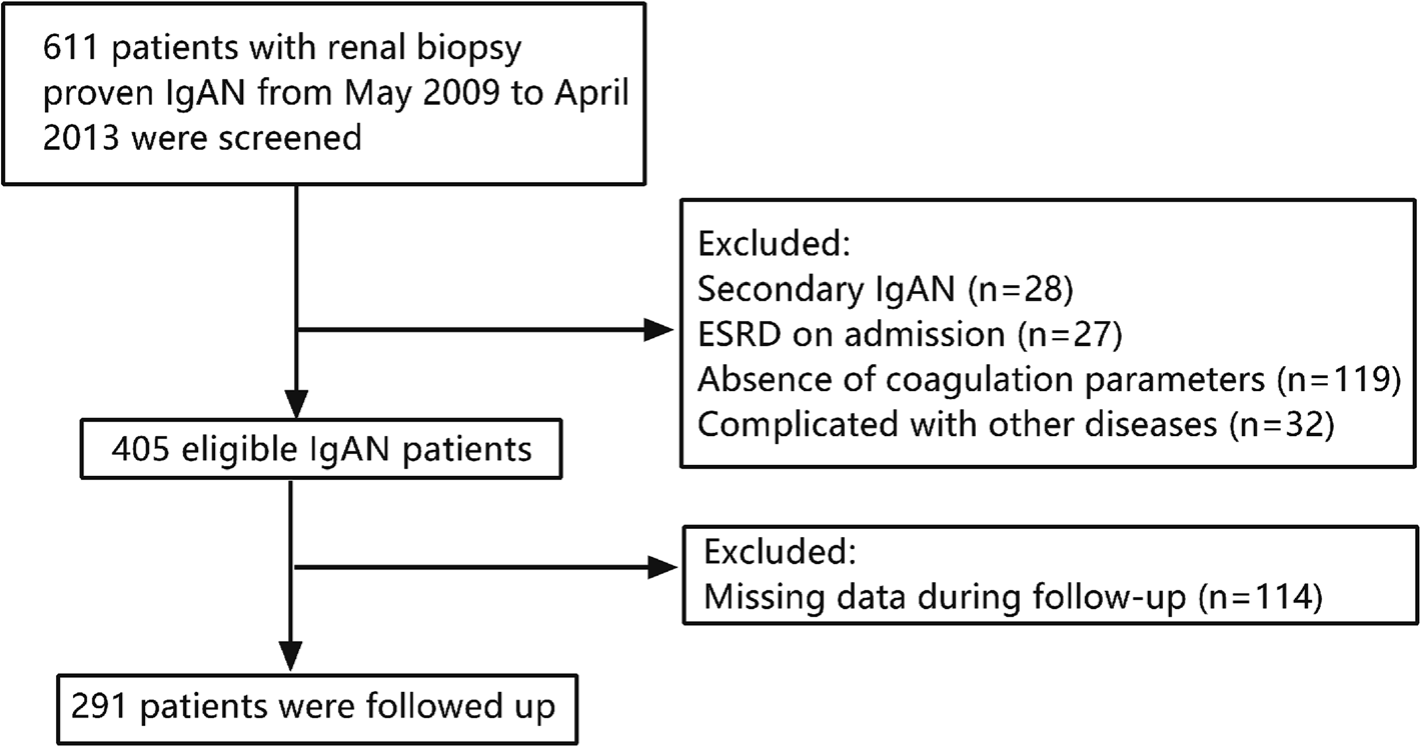
A flow diagram of the strategy used to identify IgAN patients

Two hundred and ninety-one patients with a biopsy-based diagnosis of primary IgAN between May 2009 and April 2013 were recruited at the Second Xiangya Hospital of Central South University. A flow diagram of the selection of the participants is presented in Fig. 1. The inclusion criteria were the initial estimated glomerular filtration rate (eGFR) ≥ 15 mL/min/1.73 m^2^ at the time of renal biopsy and without receiving any corticosteroids or immunosuppressants before the renal biopsy. The exclusion criteria included (1) a biopsy specimen containing less than eight glomeruli, (2) patients with secondary IgAN, such as Henoch–Schonlein purpura, lupus nephritis, hepatic disease or (3) with ESRD on admission, (4) patients with missing clinical data at the time of renal biopsy and follow-up data or (5) complicated with other diseases and taking medication that may affect coagulation function. The outcome in this study was the occurrence of ESRD or a reduction of eGFR 50% from the baseline. The study was performed with the informed consent of all patients, and the procedure was approved by the Ethics Committee of the Second Xiangya Hospital, Central South University, and is in accordance with the principles of the Declaration of Helsinki.

### Clinical and pathological measures

The following parameters were collected at baseline for each patient: age, gender, systolic blood pressure and mean arterial pressure (SBP and MAP respectively) serum creatinine (Scr), eGFR (modified Modification of Diet in Renal Disease equation) [14], 24 h proteinuria, coagulation parameters including activated partial thromboplastin time (APTT), prothrombin time (PT), thrombin time (TT), fibrinogen degradation products (FDP), fibrinogen (FIB), D-dimer (D-D), antithrombin III antibodies (AT-III).

All Renal biopsies were scored according to the new Oxford classification of the IgAN scoring system (MEST-C scoring system) proposed by the IgA Nephropathy Classification Working Group and two pathological doctors confirmed the pathological results [4]. The histologic classification was performed as follows: M0 or M1 indicated the mesangial score ≤ 0.5 or > 0.5, respectively; E0 and E1 indicated the absence or presence of endocapillary hypercellularity, respectively; S0 or S1 indicated the absence or presence of segmental glomerulosclerosis, respectively; T0, T1, and T2 indicated the degree of tubular atrophy or interstitial fibrosis (< 25%, 25–50%, and > 50%, respectively). C0, C1, and C2 indicated no crescent in glomeruli, crescent in < 25% of glomeruli, and crescent in > 25% of glomeruli, respectively.

### Statistical analysis

The numerical variables are expressed as the means with standard deviations (SD) or medians with interquartile ranges (IQR), and the categorical variables are expressed as count (%). The Chi-square test was adopted for analyzes of categorical variables and continuous variables were compared by using Student’s t-test or Kruskal-Wallis tests. The coagulation parameters cut-off values were determined by the receiver operating characteristic (ROC) curve. The Kaplan-Meier survival analysis was performed to compare the survival rate by using the log-rank test. Cox proportional hazards regression was used to perform univariate and multivariate analyses to identify prognostic factors and estimate the hazard ratios (HRs) and 95% confidence intervals (95% CIs). Binary logistic regression was used to explore the effect on the progression of IgAN in different groups. *P*-value < 0.05 was considered statistically significant. The analyses were performed using SPSS statistics 22.0 software (SPSS Inc., Chicago, IL, USA) and Graphed Prism 6 (GraphPad Software Inc., San Diego, CA, USA).

## Results

### Baseline characteristics of participants

A total of 611 biopsy-proven IgAN patients between May 2009 and April 2013 were initially screened in the study, and 291 patients were enrolled in this retrospective cohort study according to the inclusion and exclusion criteria (shown in Fig. 1). The median follow-up time was 41.2 months. Patients were divided into two groups based on the T score, and the baseline characteristics and histopathological features at the time of renal biopsy were shown in Table 1. Of all 291 patients, 131 (45.02%) were males, and the mean age was 32.50 ± 11.94 years. The mean SBP, MAP, Scr, eGFR and 24 h urine protein were 127.18 ± 11.95 mmHg, 96.3 ± 12.75 mmHg, 92.73 ± 47.14 μmol/L, 90.87 ± 69.14 mL/min/1.73 m^2^, 1.71 ± 2.51 g/day, respectively. Regarding MEST-C oxford scores in all patients, 6.53% were M1, 11% were E1, 66.67% were S1, 40.2% T ≥ 1, and 21.99% C ≥ 1. Compared to the absence of T lesion group, IgAN patients with T had significantly higher levels of SBP, MAP (129.17 ± 16.21 mmHg vs 125.84 ± 16.05 mmHg, and 98.40 ± 12.97 mmHg vs 94.90 ± 12.43 mmHg), Scr (115.35 ± 59.31 μmol/L vs 77.52 ± 28.05 μmol/L), and lower eGFR (80.1 ± 102.43 mL/min/1.73 m^2^ vs 98.11 ± 29.11 mL/min/1.73 m^2^). There was also a tendency of difference in 24 h proteinuria between two groups 1.62 ± 2.20 g/24 h vs 1.77 ± 2.71 g/24 h). For renal biopsy, 6 (5.13%) patients had M1, 15 (12.82%) had E1, 100 (85.47%) had S1, 30 (25.64%) had C1 or C2 in presence of T group. 13 (7.47%) of absence T patients had M1, 17 (9.77%) had E1, 94 (54.02%) had S1, 34 (19.54%) had C1 or C2, and percentage of S1 was significantly lower compared to the presence of T group. There were no significant differences in age, gender, M, E, C scores between the two groups.

**Table 1:** Clinical characteristics and histopathological features of study participants with and without tubular atrophy/interstitial fibrosis (T)

Characteristics	All participants (***n*** = 291)	IgAN without T (n = 174)	IgAN with T (***n*** = 117)	***p*** value
MAP (mmHg)	96.30 (12.75)	94.90 (12.43)	98.40 (12.97)	**0.023**
Scr (μmol/L)	92.73 (47.14)	77.52 (28.05)	115.35 (59.31)	**< 0.001**
eGFR (mL/min/1.73m^2^)	90.87 (69.14)	98.11 (29.11)	80.10 (102.43)	**< 0.001**
Proteinuria (g/24 h)	1.71 (2.51)	1.77 (2.71)	1.62 (2.20)	0.056
**Oxford classification**				
M0/M1 (% of M1)	272/19 (6.53)	161/13 (7.47)	111/6 (5.13)	0.428
E0/E1 (% of E1)	259/32 (11.00)	157/17 (9.77)	102/15 (12.82)	0.415
S0/S1 (% of S1)	97/194 (66.67)	80/94 (54.02)	17/100 (85.47)	**< 0.001**
T0/T1/T2 (% of [T1 + T2])	174/93/24 (40.2)	–	–	–
C0/C1/C2 (% of [C1 + C2])	227/56/8 (21.99)	140/30/4 (19.54)	87/26/4 (25.64)	0.46

The data were presented as mean (SD) or count (percentage). *Abbreviations*: *SBP* systolic blood pressure, *MAP* mean arterial pressure, *Scr* serum creatinine, *eGFR* estimated glomerular filtration rate. *P* values in bold denote significance at the 0.05 level

### Relationships between coagulation parameters and T score

**Fig. 2:**
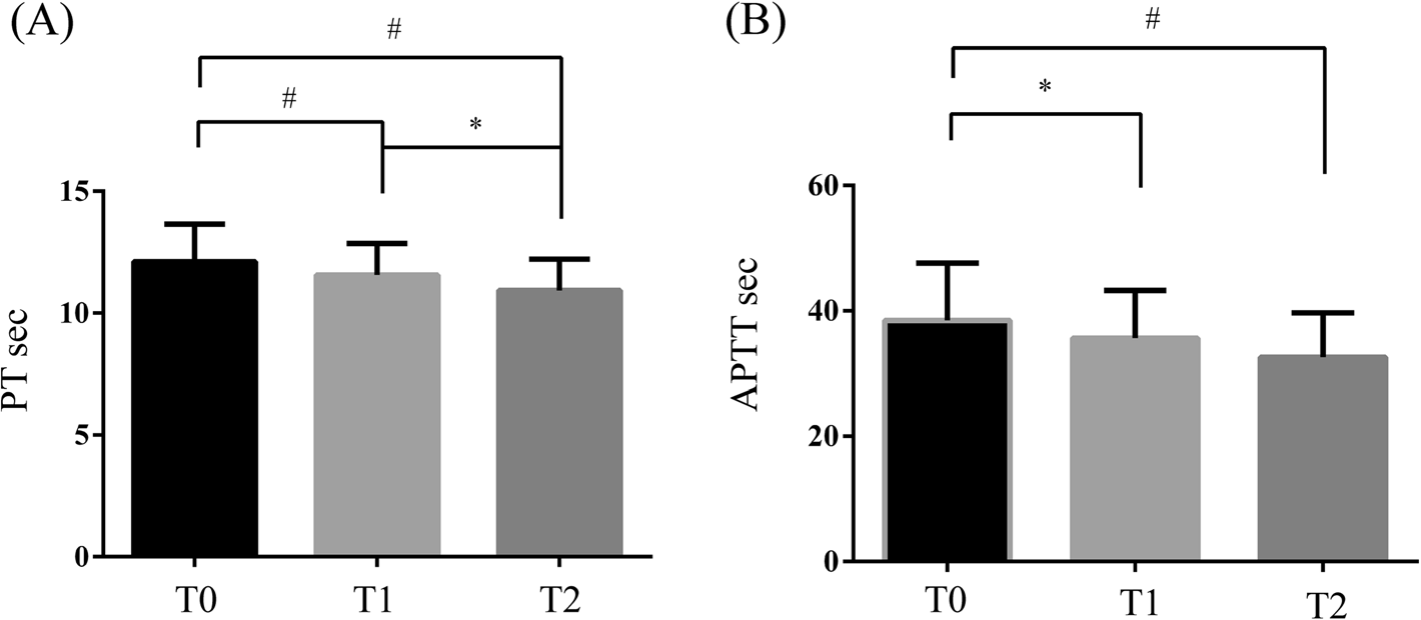
Comparison of (**a**) PT and (**b**) APTT among T0, T1 and T2 IgAN patients. T0 group (*n* = 174), T1 group (*n* = 93) and T2 group (*n* = 24). The data were presented as mean ± SD. * *p* < 0.05, # *p* < 0.01

The association of coagulation parameters, including PT, APTT, TT, FDP, FIB, D-D, AT-III, and T lesion were investigated. For all IgAN patients, 21 patients had shorter PT seconds and 16 patients had longer seconds than the reference value. For APTT, 41 of IgAN patients were shorter and 46 patients were longer than the reference. There were 22 patients with both abnormal PT and APTT levels. As is shown in Table 2, PT and APTT seconds of patients with T was significantly shorter (both *p* < 0.001) than that of the without T group. There were no statistical differences in TT, FDP, FIB, D-D, and AT-III levels. We also explored the relationship between PT, APTT, and other oxford classification of IgAN, but no significant differences were found. In addition, we would like to know whether PT and APTT are related to the degree of T rating. In the comparison of the T0 group, PT and APTT were significantly shorter in T1 (*p* = 0.003 and *p* = 0.012, respectively) and T2 group (*p* < 0.001 and p = 0.003, respectively). Moreover, PT also showed a statistical difference between T1 and T2 group (*p* = 0.036) (shown in Fig. 2).

**Table 2:** Comparison of coagulation parameters in IgAN patients without or with tubular atrophy/interstitial fibrosis (T)

Factors	All participants (n = 291)	IgAN without T (n = 174)	IgAN with T (n = 117)	p value
PT (s)	11.80 (10.80–12.70)	12.00 (11.10–12.80)	11.40 (10.45–12.30)	**< 0.001**
APTT (s)	36.40 (31.00–41.80)	38.60 (32.38–43.83)	35.20 (29.15–39.25)	**< 0.001**
TT (s)	16.80 (15.50–18.60)	16.75 (15.40–18.20)	17.10 (15.55–19.40)	0.075
FDP (μg/ml)	1.50 (1.00–2.30)	1.50 (1.00–2.50)	1.40 (1.00–2.00)	0.3
FIB (g/l)	3.07 (2.54–3.87)	3.03 (2.54–3.92)	3.10 (2.53–3.84)	0.964
D-D (μg/ml)	0.44 (0.25–0.81)	0.40 (0.23–0.83)	0.50 (0.30–0.77)	0.188
AT-III (%)	98.90 (87.00–111.00)	98.20 (85.45–109.28)	100.10 (88.65–113.50)	0.089

The data were presented as median (IQR). *Abbreviations*: *PT* prothrombin time, *APTT* activated partial thromboplastin time, *TT* thrombin time, *FDP* fibrinogen degradation products, *FIB* fibrinogen, *D-D* D-dimer, *AT-III* antithrombin III antibodies. P values in bold denote significance at the 0.05 level

### Relationships between PT, APTT, and renal outcome

**Fig. 3:**
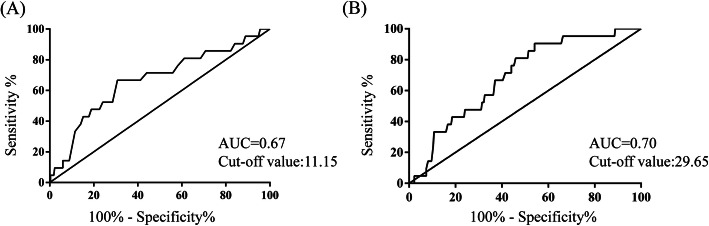
ROC curves of (**a**) PT, (**b**) APTT for the identification of poor renal outcome. The best cut-off value for PT and APTT are labeled on the plot

**Fig. 4:**
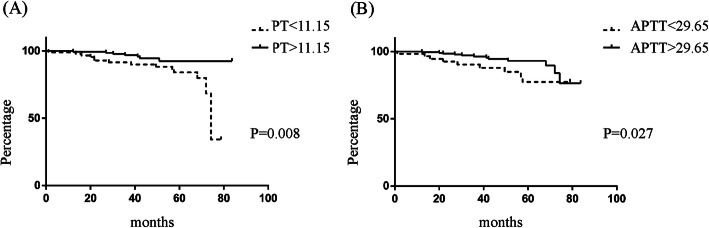
Kaplan–Meier curves of ESRD/50% drop in eGFR from baseline according to (**a**) PT, (**b**) APTT levels category

Of all the participants, a 50% decline in eGFR was observed in 4.8% (*n* = 14) of the patients, and 2.4% (*n* = 7) of the patients developed ESRD during the follow-up period. In order to investigate the association of PT, APTT, and renal progression, ROC curves were firstly constructed (shown in Fig. 3). The areas under the ROC curves (AUCs) for PT and APTT were 0.67 (95%CI 0.54–0.79) and 0.7 (95%CI 0.59–0.79). Based on PT and APTT cut-off value, patients were divided into PT < 11.15 s and > 11.15 s groups, or APTT< 29.65 s and > 29.65 s groups respectively. Kaplan-Meier survival analysis was performed to examine renal survival between groups (shown in Fig. 4). The cumulative survival rate was significantly higher in patients with longer PT (> 11.15 s) or APTT (> 29.65 s) than the lower groups (*p* = 0.008, *p* = 0.027 respectively).

Cox proportional hazards models were constructed to determine the prognostic value of PT, APTT, and clinical/histopathological parameters in IgAN patients (Table 3). Univariate analysis showed higher values of Scr (HR 1.22, 95%CI 1.16–1.28, per 10umol/L increase), SBP (HR 1.34, 95% CI 1.08–1.67, per 10 mmHg increase), 24 h proteinuria (HR 1.13, 95%CI 1.00–1.28, per 1 g increase) and lower values of eGFR (HR 0.52, 95%CI 0.41–0.66, per 1 mL/min/1.73 m2 increase), PT (HR 3.27, 95%CI 1.30–8.24 versus > 11.15 s group), APTT (HR 2.59, 95%CI 1.08–6.21 versus > 29.65 s group) at baseline were significantly associated with a higher risk of renal outcome. The biopsy score S1 (HR 3.68, 95% CI 1.08–12.59) and T ≥ 1 (HR 13.16, 95%CI 3.06–56.67) also significantly correlated to poor prognosis. However, after adjusting for univariate risk factors, the multivariate analysis showed no significant value in PT or APTT (data not shown).

**Table 3:** Association between baseline characteristics of patients and the incidence of renal outcome

Baseline variables	HR	95% Lower	95% Upper	p value
**Demographics**				
Age, per 10 year	0.81	0.55	1.20	0.29
Gender (male versus female)	0.98	0.41	2.33	0.964
**Laboratory measures at baseline**				
Scr, per 10 umol/L	1.22	1.16	1.28	**< 0.001**
eGFR, per 10 mL/min/1.73m^2^	0.52	0.41	0.66	**< 0.001**
SBP, per 10 mmHg	1.34	1.08	1.67	**0.007**
24 h proteinuria, per 1 g	1.13	1.00	1.28	**0.05**
PT (< 11.15 s versus > 11.15 s)	3.27	1.30	8.24	**0.012**
APTT (< 29.65 s versus > 29.65 s)	2.59	1.08	6.21	**0.032**
**Histology at biopsy**				
M (1 versus 0)	0.05	0.00	223.16	0.476
E (1 versus 0)	0.79	0.18	3.41	0.752
S (1 versus 0)	3.68	1.08	12.59	**0.038**
T (≥1 versus 0)	13.16	3.06	56.67	**0.001**
C (≥1 versus 0)	1.58	0.61	4.10	0.344

*Abbreviations*: *SBP* systolic blood pressure, *Scr* serum creatinine, *eGFR* estimated glomerular filtration rate. P values in bold denote significance at the 0.05 level

**Fig. 5:**
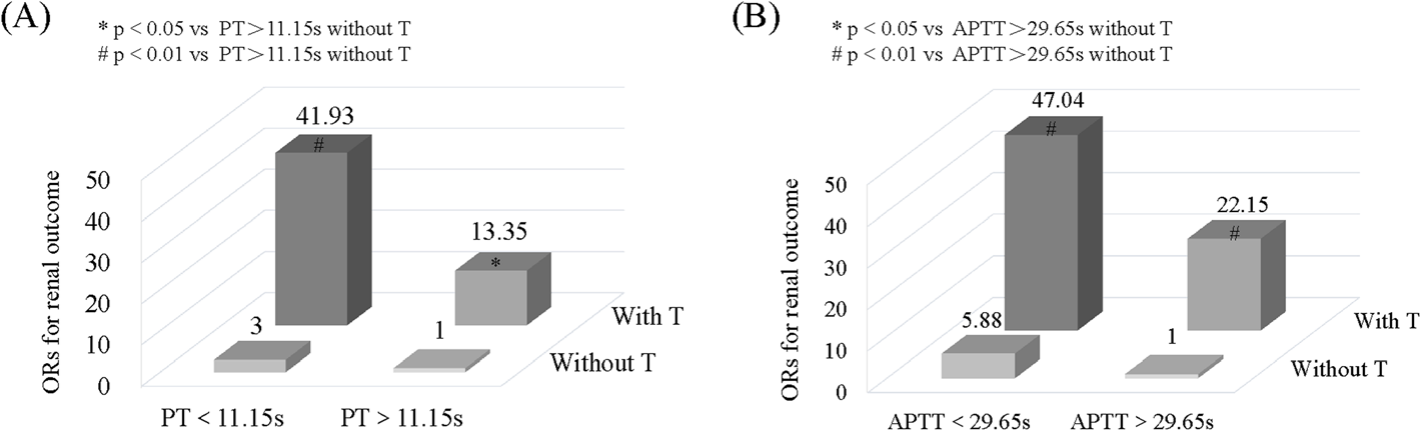
The analysis of the additive effect of (**a**) PT, (**b**) APTT, and T score on the renal outcome. The renal outcome was defined as an ESRD or an irreversible 50% eGFR reduction. X-axis stood for subgroups of PT or APTT. Z-axis stood for with/without T, and the Y-axis stood for the OR value. Patients without T and PT > 11.15 s or APTT> 29.65 s were set as the reference, respectively. * p < 0.05, # p < 0.01

Thus, we assessed the additive effect of PT or APTT and T lesion on the renal outcome (shown in Fig. 5). Patients without T lesion and with PT > 11.15 s or APTT> 29.65 s were set as the reference groups. The odd ratios (ORs) were 3 (95%CI: 0.18–49.00; *P* = 0.44) for patients without T and PT < 11.15 s, 13.35 (95%CI: 1.57–113.37; *P* = 0.018) for patients with T and PT > 11.15 s, and 41.93 (95%CI: 5.32–330.48; *P* < 0.001) for patients with T and PT < 11.15 s. This association was consistent with the combination of APTT and T lesion. The corresponding ORs were 5.88 (95%CI: 0.36–97.09; *P* = 0.22), 22.15 (95%CI: 2.81–174.89, *P* = 0.003), and 47.04 (95%CI: 5.64–392.55; P < 0.001).

## Discussion

Considering that T score is a well-recognized independent predictor of IgAN, it is worth exploring the possible risk factors associated with T. Studies have reported that tubulointerstitial injury of IgAN was associated with trefoil factor 3 mRNA [15], p38 MAPK activity [16], BMI [17], serum matrix metalloproteinase-7 level [18]. In this study, we demonstrated the significance of coagulation parameters PT and APTT in the tubulointerstitial injury of IgAN.

Strong evidence indicates that the components of coagulation factors may cause ischemia and localized blood flow disruption through the formation of microthrombus, and drive pro-fibrotic events [19]. Moreover, anticlotting drugs were reported to reduce fibronectin accumulation and improve renal fibrosis [20, 21]. Actually, the coagulation system is a complex balance of coagulant and anticoagulant components that functions in unique homeostasis by interacting with activated endothelial cells, coagulation factors, etc. [22]. In the kidney, when microvascular endothelial cells are abnormally damaged, the activation of platelets and the release of plasma coagulation factors can activate intra-glomerular coagulation [23]. PT, APTT, TT, FDP, FIB, D-D, and AT-III measurements are routine coagulation tests used to assess coagulation systems prior to renal biopsy. The APTT and the PT reflect the function of endogenous and exogenous pathways of the coagulation cascade [24], and its duration could be affected by the upstream Xa factor common to coagulation activation pathways. Reports suggested that factor Xa could cooperate with V to exert procoagulant activity in active IgAN, leading to intra-mesangial coagulation [25, 26]. Prothrombin/thrombin has been reported to be elevated in acute kidney injury (AKI), nephrotic syndrome, CKD and other renal diseases, inhibition could attenuate tubular atrophy or proliferative responses in renal [27]. However, no study has shed light on the relationship between coagulation parameters and clinical or pathological data in IgAN. In our study, APTT and PT were found significantly shorter (both *p* < 0.001) in IgAN patients with tubular atrophy/interstitial fibrosis (T) when compared with the absence of T group, suggesting the activation of endogenous and exogenous pathways of the coagulation cascade. This also suggests that renal fibrosis may be accompanied by a hypercoagulable state, which is consistent with the coagulation situation found in patients with pulmonary fibrosis or cirrhosis, and anticoagulation as a therapeutic option is promising [28–30].

Glomerular FIB activation is a secondary event after vascular injury in glomerular clusters. Urinary fibrinogen was shown to correlate with interstitial fibrosis and was an independent risk factor for the progression of CKD to ESRD [31]. TT reflects the ability of subjects to convert plasma FIB to fibrin [32]. In IgAN, soluble fibrin deposition was reported to be found in kidney tissue specimens in its early stage [33]. In our results, there was no significant difference in FIB and TT between IgAN with T and without groups. AT-III, D-D, FDP are indicators of the activity status of the fibrinolytic system. AT-III is an important anticoagulant in the body and participates in the dynamic balance of the coagulation and fibrinolytic systems, its administration was shown beneficial for interstitial fibrosis in AKI model [34]. In IgAN patients, we found no difference in the AT-III level between the IgAN with T group and without T group, suggesting that the anticoagulant system was not obviously altered when the presence of fibrosis. D-D and FDP are the degradation product of fibrin, both of which reflect the fibrinolytic function [35]. No difference was observed between the two groups, indicating that interstitial fibrosis/tubular atrophy was not accompanied by a hyper/lower activity of fibrinolysis. Apart from coagulation parameters, SBP, MAP, Scr, eGFR, and S score were also associated with the degree of renal interstitial fibrosis in IgAN from our results.

In our previous study, serum D-D was found to serve as a potential predictor for thrombotic events in patients with kidney disease [36]. For IgAN, Scr, eGFR, proteinuria, and pathology characteristics have been identified as baseline predictors of progression [8, 37–39], which is consistent with our findings. As coagulation function showed an association with the T score of pathology as described, and could be well monitored in a clinical laboratory, it is of clinical significance to explore the relationship between coagulation function and the prognosis of IgAN. The shorter PT (< 11.15 s) and APTT (< 29.65 s) showed a significantly worse prognosis compared to the longer group according to the Kaplan–Meier survival curve. Although univariate cox analysis of PT and APTT was significant, the multivariate analysis adjusted for univariate risk factors was not. This indicates that PT and APTT have limited ability as independent predictors but maybe secondary factors that increase the risk of poor prognosis in IgAN. The analysis of the combination of a shorter PT or APTT with T lesion could adversely affect the renal outcome more in IgAN also supports that shorter PT and APTT indirectly accelerated the progression.

It is well known that nephrotic-range proteinuria is related to acquired complex hypercoagulability involving the acceleration of the entire thrombotic process (activated intrinsic pathway, fibrinogen, platelet function and fibrin-platelet interaction) [40, 41]. Hypercoagulability was found highly correlated with the severity of nephropathy and likely secondary to nephrosis [42]. Although no clear molecular therapeutic targets have been identified, most studies have shown that the main pathological changes involve antithrombin, fibrinogen, and factors V and VIII [43]. Also, in most CKD patients, coagulation disorder is an important non-immune factor for the occurrence and disease development [44]. Correcting coagulation imbalance is of great significance for early CKD stage treatment, and direct oral anticoagulants use in patients with ESRD and advanced CKD is increasing [45]. For IgAN, there is insufficient evidence to determine the causal relationship between coagulation and disease progression. Although early studies of anticoagulation therapy showed that warfarin combined with dipyridamole was not effective in IgAN treatment [46], the combination therapy including prednisolone, mizoribine with warfarin and dipyridamole were found better for severe childhood IgAN than the combination treatment including prednisolone and mizoribine without warfarin or dipyridamole from the point of proteinuria remission [47]. This suggests that anticoagulant may be promising as an adjunctive drug in IgAN, and determine the relationship between hypercoagulability and markers of disease severity may help to identify clinically meaningful towards when and how anticoagulants warranted.

To the best of our knowledge, this is the first paper that focuses on the relationship between coagulation function, T score, and IgAN prognosis. Possible mechanisms to explain the association between shorter PT or APTT and interstitial fibrosis/tubular atrophy are as follows. (1) With the progression of IgAN, renal impairment is aggravated, renal units are damaged accompanied by fibrosis, resulting in loss of normal excretory function and reduced clearance of procoagulant substances. (2) Endothelial injury is a common manifestation in IgAN [48, 49], the shortening of PT or APTT may be related to the initiation of coagulation by vascular endothelial injury. (3) Moreover, extensive researches have found that coagulation is related to inflammatory response, and IgAN is also an inflammatory disease. IgAN patients are commonly associated with alteration of various inflammatory cytokines [50], which may be related to the activation of procoagulant factors and renal fibrosis [51, 52]. In addition, there are some limitations to our study. First, the data were collected at a single center, which may limit the generalisability of the results. Second, although those on treatment with agents affecting coagulation parameters were excluded, we did not consider treatment with angiotensin-converting enzyme inhibitors/angiotensin receptor blockers or antihypertension therapy and how this may affect the results and vascular physiology.

In conclusion, our study reveals a novel aspect of tubular atrophy/interstitial fibrosis (T), linking coagulation function to clinical and pathological of IgAN. Shorter PT and APTT are associated with the presence of T and may increase the risk of poor prognosis in IgAN. The molecular mechanism between coagulation and IgAN pathology needs to be further explored.

## Data Availability

The datasets used and/or analyzed during the current study are available from the corresponding author on reasonable request.

## Abbreviations

IgAN: Immunoglobulin A nephropathy
ESRD: End-stage renal disease
CKD: Chronic kidney disease
M: Mesangial hypercellularity
E: Endocapillary hypercellularity
S: Segmental glomerulosclerosis
T: Interstitial fibrosis/tubular atrophy
C: Crescent
eGFR: Estimated glomerular filtration rate
SBP: Systolic blood pressure
MAP: Mean arterial pressure
Scr: Serum creatinine
APTT: Activated partial thromboplastin time
PT: Prothrombin time
TT: Thrombin time
FDP: Fibrinogen degradation products
FIB: Fibrinogen
D-D: D-dimer
AT-III: Antithrombin III antibodies
SD: Standard deviations
IQR: Interquartile ranges
ROC: Receiver operating characteristic
HR: Hazard ratios
CI: Confidence intervals
AUC: Areas under the ROC curve
OR: Odd ratios
